# Advancing Knowledge of Acute Cutaneous Graft-Versus-Host Disease Wound Care: A Case Study

**DOI:** 10.1097/WON.0000000000001240

**Published:** 2026-01-10

**Authors:** Sara Errichiello, Chiara Visintini, Chiara Rosignoli, Diana Cerne, Giuseppe Petruzzellis, Francesca Patriarca, Alvisa Palese

**Affiliations:** **Sara Errichiello, RN,** Division of Haematology and Stem Cell Transplantation, Clinical University Hospital “Santa Maria della Misericordia”, Udine, Italy.; **Chiara Visintini, PhD, RN,** Division of Haematology and Stem Cell Transplantation, Clinical University Hospital “Santa Maria della Misericordia”, Udine, Italy.; **Chiara Rosignoli, MD,** Department of Pediatric Onco-Hematology Cell and Gene Therapy, Ospedale Pediatrico “Bambino Gesù”, Rome, Italy.; **Diana Cerne, MNS, RN,** Integrated Area of Oncology and Radiotherapy, Clinical University Hospital “Santa Maria della Misericordia”, Udine, Italy.; **Giuseppe Petruzzellis, MD,** Division of Haematology and Stem Cell Transplantation, Clinical University Hospital “Santa Maria della Misericordia”, Udine, Italy.; **Francesca Patriarca, PhD, MD,** Division of Haematology and Stem Cell Transplantation, Clinical University Hospital “Santa Maria della Misericordia”, Udine, Italy.; **Alvisa Palese, PhD, RN,** School of Nursing, Department of Medicine, University of Udine, Italy.

**Keywords:** Acute cutaneous graft-versus-host disease, Allogeneic hematopoietic stem cell transplantation, Nursing, Soft silicone layer, Wound care

## Abstract

**BACKGROUND:**

Acute cutaneous graft-versus-host disease (GvHD) is a frequent complication of patients undergoing allogeneic hematopoietic stem cell transplantation (HSCT). It requires multidisciplinary systemic and topical management. However, no well-established recommendations regarding wound management have been established. This case study describes our experience caring for the wound of a patient with acute myeloid leukemia who developed a grade IV stage 4 acute cutaneous GvHD.

**CASE:**

Mr T was a 64-year-old male diagnosed with acute myeloid leukemia with myelodysplasia-related changes. Seventy-five days after receiving an allogeneic human leukocyte antigen-HSCT, he developed wounds on his upper and lower limbs, part of his chest, torso, and sacrum. Four lines of therapy (systemic steroids, tacrolimus together with extracorporeal photopheresis, etanercept, and ruxolitinib) integrated with wound care using advanced dressings of soft silicone layers led to the resolution of his wounds on day 109.

**CONCLUSION:**

Our case study provides further evidence for the management of cutaneous GvHD. Further studies are required to assess the effectiveness of silicone layer dressings for managing allogeneic HSCT recipients with cutaneous GvHD on a large scale to provide definitive recommendations for their use.

## INTRODUCTION

One of the most prevalent complications of hematopoietic stem cell transplantation (HSCT) is an acute graft-versus-host disease (GvHD). This complication tends to occur in the immediate post-transplant period; reported incidence rates vary from 30% to 50%.^1^,^2^ The occurrence of a GvHD reduces health-related quality of life and graft survival.^3^,^4^ The staging system of acute GvHD is based on the involvement of the skin, the upper and lower gastrointestinal (GI) tract, and the liver; clinical grades range from 0 to IV based on the severity of the involvement of these organs.^5^ Grade 0 is no stage 1 to 4 of any organ. Grade I is stage 1 to 2 skin without liver or upper or lower GI involvement. Grade II is stage 3 rash and/or stage 1 liver and/or stage 1 upper GI and/or stage 1 lower GI. Grade III is stage 2 to 3 liver and/or stage 2 to 3 lower GI, with stage 0 to 3 skin and/or stage 0 to 1 upper GI. Grade IV is stage 4 skin, liver, or lower GI involvement, with stage 0 to 1 upper GI (Table 1).^5^

**TABLE 1.** Staging of Acute GvHD According to the Classification of Harris et al^5^

**Stage**	**Skin**
0	No active (erythematous) GvHD rash
1	Maculopapular rash <25% BSA
2	Maculopapular rash 25%%-50% BSA
3	Maculopapular rash >50% BSA
4	Generalized erythroderma (>50% BSA) plus bullous formation and desquamation >5% BSA
**Stage**	**Liver (bilirubin)**
0	<2 mg/dL
1	2-3 mg/dL
2	3.1-6 mg/dL
3	6.1-15 mg/dL
4	> 15 mg/dL
**Stage**	**Upper GI**
0	No or intermittent nausea, vomiting, or anorexia
1	Persistent nausea, vomiting, or anorexia
**Stage**	**Lower GI (stool output/day)**
0	<500 mL/day or <3 episodes/day
1	500-999 mL/day or 3-4 episodes/day
2	1000-1500 mL/day or 5-7 episodes/day
3	>1500 mL/day or >7 episodes/day
4	Severe abdominal pain with or without ileus or grossly bloody stool (regardless of stool volume)
**Overall clinical grade**	**Description (based on most severe target organ involvement)**
0	No stage 1-4 of any organ
I	Stage 1-2 skin without liver, upper GI, or lower GI involvement
II	Stage 3 rash and/or stage 1 liver and/or stage 1 upper GI and/or stage 1 lower GI
III	Stage 2-3 liver and/or stage 2-3 lower GI, with stage 0-3 skin and/or stage 0-1 upper GI
IV	Stage 4 skin, liver, or lower GI involvement, with stage 0-1 upper GI

Abbreviations: BSA, body surface area; GI, gastrointestinal.

The 5 stages for cutaneous manifestations refer to the body surface area (BSA) involved by a maculopapular rash: stage 0 is no active rash, stage 1 is a maculopapular rash on <25% of the BSA, stage 2 is a maculopapular rash on 25% to 50% of BSA, stage 3 is a maculopapular rash on >50% of BSA, and stage 4 is generalized erythroderma (>50% BSA) with bullae formation and desquamation >5% BSA. The diagnosis is basically clinical.^5^

Acute cutaneous GvHD is based on a physiopathology mechanism triggered when the immune thymus cells (T cells) in the donated tissue (the graft) recognize the recipient (the host) as a stranger; the resulting immune response activates donor T cells by promoting cytolytic activity, which attacks the recipient’s tissues to eliminate foreign antigens.^6^ Human leucocyte antigen (HLA) disparity, advanced age of donors or recipients, donor’s alloimmunization (eg, previous transfusions, pregnancy), use of peripheral blood as a source of hematopoietic stem cells, and intensity of the conditioning regimen have all been identified as acute GvHD risk factors.^6^,^7^

Interventions are based on continuous assessment of cutaneous manifestations, management of itchy and painful associated symptoms, dosage of immunosuppressants, application of topical and systemic medications,^8^ and emotional support.^9^ However, despite its dramatic impact on patients and the risk of infection, it is still unclear how to manage wounds associated with acute cutaneous GvHD; differences in management between bone marrow transplant centers (BMTCs) have been reported and are often based on anecdotal experiences.^9^ The available literature recommends the topical use of calcineurin inhibitors for the treatment of chronic cutaneous GvHD^10^ or the application of hydrocolloid-impregnated polyurethane films for burn wounds.^11^ However, to the best of our knowledge, evidence on acute cutaneous GvHD only suggests the application of topical products such as corticosteroid ointments for stages 1 and 2^12^,^13^; and no recommendations for wound care are available for higher stages.

In this context, we report the case of Mr T with acute cutaneous GvHD grade IV stage 4 hospitalized in our BMTC who was treated with systemic therapy and advanced dressings with a soft silicone layer.

## CASE

Mr T was a 64-year-old male diagnosed with acute myeloid leukemia with myelodysplasia-related changes. He had a past medical and surgical history of anxiety-depressive syndrome, arterial hypertension, thyroidectomy, bilateral cataracts, hemorrhoidal pathology, gastroesophageal reflux disease, polycystic kidney, and a normal body mass index of 18.5. He was previously admitted to a hospital for standard treatment for acute myeloid leukemia where he received 4 cycles of chemotherapy with complete remission.

He was admitted to our BMTC to receive a sibling HLA-identical HSCT. The myeloablative conditioning regimen given prior to HSCT consisted of the administration of busulfan (Busulfex Tillomed, Tillomed Laboratories Ltd, Luton, United Kingdom) 3.2 mg/kg/day, intravenously, 4 times a day for 4 days and fludarabine (Fludarabina Teva, Teva Italia, Milan, Italy) 40 mg/m^2^, intravenously, once daily for 3 days through a central venous catheter.

The prophylaxis of GvHD consisted of the administration of rabbit anti-thymocyte immunoglobulin (Grafalon, Neovii Pharmaceuticals AG, Rapperswil-Jona, Switzerland) 10 mg/kg, intravenously, once daily for 3 days prior to HSCT, cyclosporine (Sandimmun, Novartis Farma, Milan, Italy) intravenously, twice daily, from the day before HSCT, starting at 3 mg/kg/day and adjusting it according to therapeutic drug monitoring until 39 days post HSCT. Methotrexate (Metotrexato Teva, Teva Italia, Milan, Italy) 15 mg/m^2^ once daily was administered intravenously on the first day after HSCT and 10 mg/m^2^ once daily, and on the third and sixth days after HSCT. In addition, Pretransplant rituximab (Truxima, Mundipharma Pharmaceuticals, Milan, Italy) 360 mg intravenously was administered the day before HSCT. The next day, Mr T received CD34 + hematopoietic stem cells (8.5 × 10^6^/kg) and CD3+ (21 × 10^7^/kg) from peripheral blood of the HLA-matched sibling donor, homogroup AB0. Granulocyte recovery (neutrophils >1000/mmc) was recorded on the 15th day post HSCT.

Seventeen days post HSCT, he presented with facial, trunk, bilateral palms of hands and bilateral lower limb erythema and pruritis, leading to the diagnosis of acute cutaneous GvHD grade II stage 3 (maculopapular rash >50% of BSA), without liver or GI involvement. Therefore, the first-line therapy of acute GvHD was initiated with the administration of 6-metilprednisolone (Urbason, Sanofi Italia, Milan, Italy) at 2 mg/kg/day intravenously from the 17th day post HSCT, followed by a slow tapering phase until it was discontinued at 33 days post HSCT, with signs of cutaneous improvement. At 38 days post HSCT, he had dystrophic skin with multiple crusty wounds on the elbows, lower limbs, and abdomen, which were treated with topical almond oil twice a day (galenic preparation from our hospital pharmacy, bottle of 50 mL). It was a galenic product; thus, it did not have a brand name. At 39 days post HSCT, with the diagnosis of T thrombotic microangiopathy (TMA), 6-metilprednisolone at 1 mg/kg daily was restarted intravenously and cyclosporine was discontinued. Moreover, a topical steroid therapy based on hydrocortisone-17-butyrate 0.1% (Locoidon, Leo Pharma, Rome, Italy) was applied twice a day for 8 days, with no signs of improvement.

Forty-seven days post HSCT, TMA was improving while the wounds were worsening; therefore, a second-line therapy with an inhibitor of calcineurin, tacrolimus (Prograf, Astellas Pharma, Milan, Italy) 0.5 mg intravenously daily was initiated in addition to weekly sessions of extracorporeal photopheresis (ECP). At 70 days post HSCT, almost 1 month after the beginning of the second-line therapy and despite some encouraging signs of cutaneous GvHD response, tacrolimus was discontinued secondary to TMA. The concomitant administration of etanercept (Enbrel, Pfizer, Puurs-Sint-Amands, Belgium), a tumor necrosis factor-α inhibitor, 50 mg subcutaneously, twice a week was added as a third-line therapy at 70 days post HSCT.

**Figure 1.**
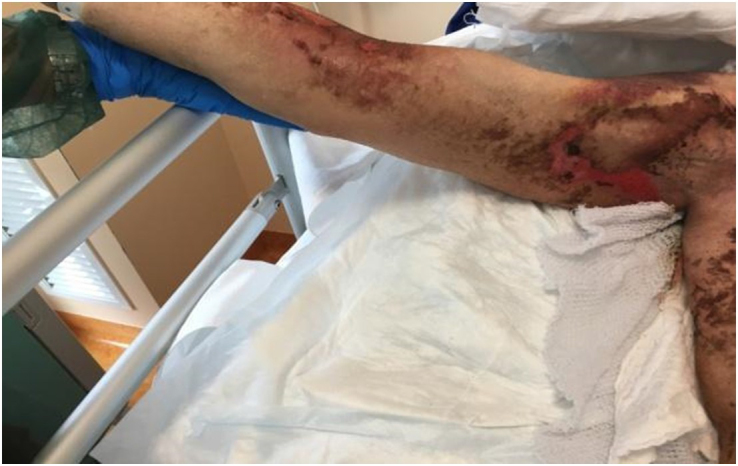
Acute cutaneous GvHD grade IV stage 4 with areas of desquamation at the right upper limb (at 75 days post HSCT).

However, at 75 days post HSCT, GvHD was no more responsive and worsened to grade IV stage 4 (generalized erythroderma (>50% BSA) plus bullous formation and desquamation >5% BSA and no liver or GI involvement) with extensive painful cutaneous wounds at the upper and lower limbs, part of the chest and torso, and also at the sacral level (Figure 1).

Consequently, a fourth-line off-label therapy with JAK 1 to 2 inhibitor ruxolitinib (Jakavi, Novartis Pharma, Milan, Italy) was initiated orally at 10 mg twice a day, and then adjusted according to the blood count and the GvHD response. Concurrently, the administration of etanercept was discontinued.

Skin care including recommended hygiene care, careful drying, and special clothing^8^ was initiated in addition to systemic therapy implemented from the 70th day post HSCT as described earlier. Nurses performed gentle hygiene care with warm water and a moisturizing cleanser (Bioderm Dermolatte, Farmoderm, Novate Milanese, Italy) once a day for the treatment of dry and sensitive skin, with a self-balancing pH, carefully drying the skin without rubbing, to avoid new micro-traumas to the skin; postural changes were initiated every 2 hours to keep pressure off his wounds, and he was encouraged to wear natural and nonsynthetic fiber pajamas and underwear; appropriate nutrition and hydration as advised by the dietitian included drinking 2 L of water daily and increasing daily requirements by 30%, with the intake of high-calorie foods.

When the wounds began to bleed superficially and uniformly on almost his entire body surface (upper and lower limbs, part of the chest, torso, and sacral level) from the 75th day post HSCT, they were cleansed with lactated Ringer’s solution (Ringer Lattato, Fresenius Kabi Italia, Verona, Italy) preheated to a temperature between 37°C and 39°C. Drying was achieved by gently dabbing without rubbing to reduce further damage to the wound beds. Care was provided daily with the use of sterile gloves, gauze, and drapes to reduce the infection risk; moreover, Mr T was covered during dressing changes to neutralize the cold sensation caused by the loss of thermoregulatory function related to the detachment of the epidermal layer. A continuous assessment of pain before and after each dressing change was performed; oral morphine sulfate 10 mg/5 mL (Oramorph, Molteni Farmaceutici, Scandicci, Italy) was administered before each treatment.

**Figure 2.**
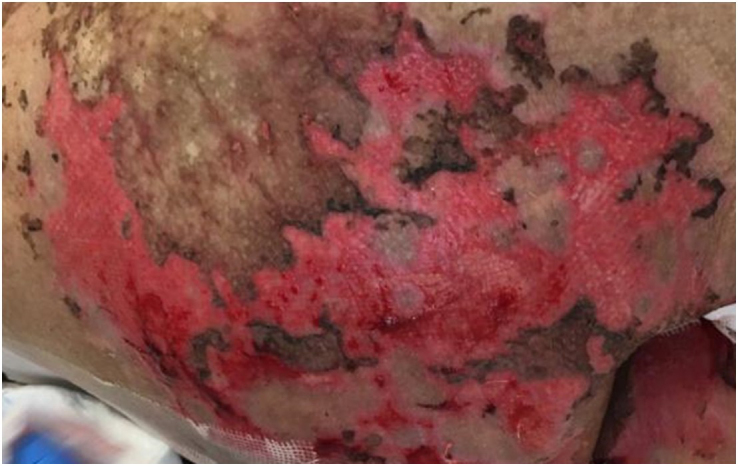
Acute cutaneous GvHD grade IV stage 4 with desquamation at the sacral level (at 75 days post HSCT).

An advanced dressing of soft silicone in the form of a polyester mesh with a very thin micro-perforated silicone layer (Mepitel One, Mölnlycke Health Care, Göteborg, Sweden) was applied to the superficial and exudating wounds on the upper limbs, part of the chest, torso, and sacral level once daily, in the morning, starting from the 75th day post HSCT (Figure 2). Wounds with fibrin mainly on the upper and lower limbs were treated with hydrogel with alginate (Nu-Gel, 3M GmbH, Rüschlikon, Switzerland), which was applied under the silicone layer. This second dressing was replaced every 2 days.

The silicone dressing was then covered with laparotomy gauze (Tampone laparotomico di garza idrofila, Mediberg, Bergamo, Italy), which allowed the coverage of a greater body surface and was secured with a tubular net (Bendelast, Pharmac-Zabban, Calderara di Reno, Italy), both at the torso and at the legs. The use of adhesive dressings was avoided to prevent skin tears.

**Figure 3.**
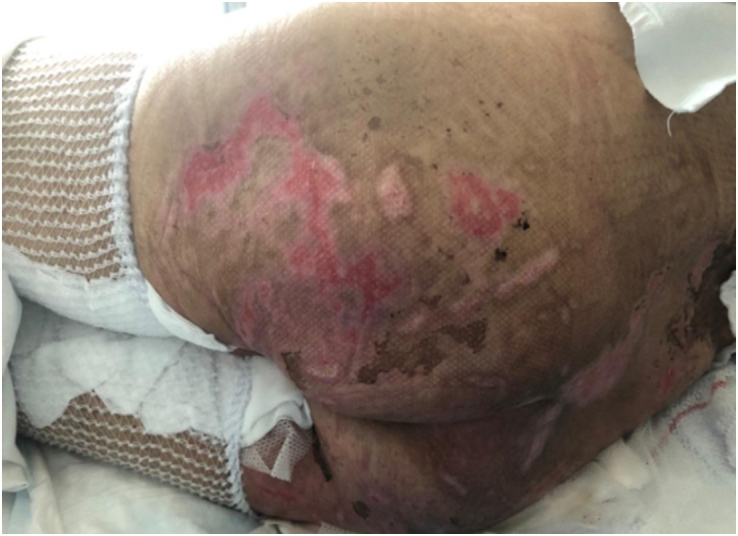
Initial re-epithelialization at the sacral level (at 83 days post HSCT).

In 4 days (79 days post HSCT), the wounds were less exudative with less bleeding, with a slight improvement of the cutaneous GvHD, while the wounds with fibrin had less fibrin and a more granulation tissue; after the ninth session of ECP (83 days post HSCT), there was an initial re-epithelialization of all the wounds, both the exudating and the wounds with fibrin at the sacral level (Figure 3), with no new wounds.

Eighty-nine days post HSCT, the dressings were changed every other day and transparent polyurethane dressings (Tegaderm roll, 3M GmbH, Rüschlikon, Switzerland) were also used to stabilize the laparotomy gauze.

**Figure 4.**
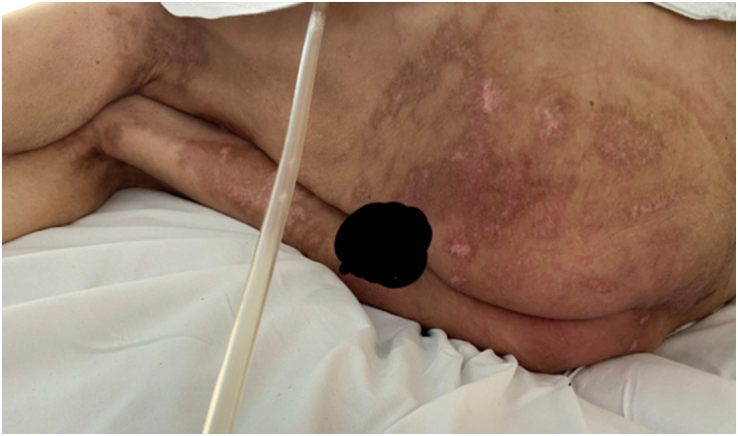
Complete resolution of acute cutaneous GvHD at the sacral level (at 109 days post HSCT).

**Figure 5.**
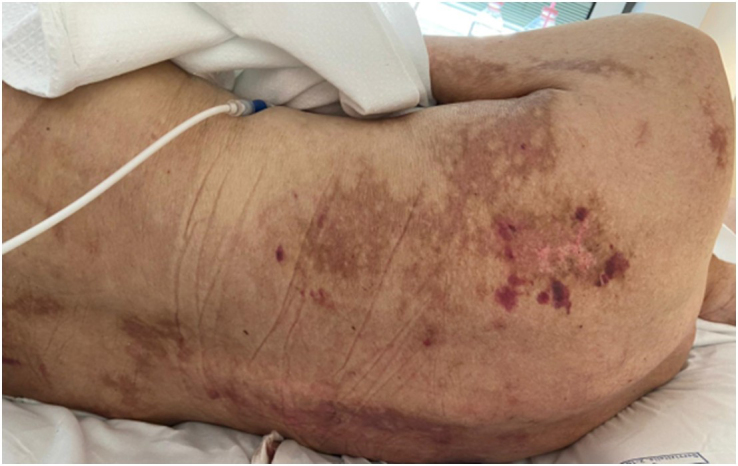
Complete resolution of acute cutaneous GvHD to upper limbs and torso (at 109 days post HSCT).

On the 104th day post HSCT, there was resolution of all wounds except for 3 small wounds on Mr T’s back (2.0 × 2.0 cm), which were treated with silicone dressings. After a few days, GvHD was considered resolved (Figures 4 and 5). Mr T received 10 sessions of ECP, along with the administration of daily oral ruxolitinib which was continued after hospital discharge.

## DISCUSSION

To date, wound care in acute cutaneous GvHD is mostly based on anecdotal approaches^9^ and primarily determined by patient presentation, symptoms, and preferences.^14^ Dressings used for burns may be used given the analogy between stages 3 and 4 GvHD wounds and II and III grade burn injuries.^8^,^15^ Silver sulfadiazine ointment,^16^ polyurethane foam dressings containing silver,^11^ and hydrocolloid-impregnated polyurethane films, dispersed in a lipid matrix,^17^ have been reported and evaluated in the healing of burn wounds. A randomized controlled trial by David and colleagues^18^ among patients with traumatic injuries and burns compared a dressing with polyurethane film with a layer of soft silicone contact to polyurethane film impregnated with hydrocolloid dispersed in a lipid matrix. Results revealed that both medications were well tolerated.^18^ Finally, the use of a hydrogel and an antibacterial protease containing lysostaphin dressing was applied in a 53-year-old man with ulcers on the right leg with promising results; however, the patient was affected by chronic GvHD.^19^

We chose to use an advanced dressing based on a soft silicone layer. Among patients with traumatic injuries and burns, the comparison of a polyurethane film with a layer of soft silicone contact to a polyurethane film impregnated with hydrocolloids showed better findings for the soft silicone layer in terms of wound healing and painless removal.^18^ In a case series of 3 patients with epidermolysis bullosa, a soft silicone dressing was found to be effective in promoting wound healing.^20^ In a case series of 2 patients with partial thickness wounds, a nonadhesive silicone net dressing was reported to result in a fast-healing process, absorbing the drainage of exudate from the wound bed in an atraumatic manner.^21^ Among patients with skin tears, the use of a silicone adhesive layer reduced the pain and discomfort during the dressing changes when compared to traditional dressings.^22^,^23^ In general, soft silicone dressings can remain in place for several days, thus ensuring an undisturbed healing process.^24^,^25^

A few days after the use of a soft silicone layer dressing, Mr T’s wounds revealed signs of improvement, with complete healing after 1 month of treatment. To the best of our knowledge, only 1 case reported in the field of acute GvHD has been published to date regarding a 64-year-old man undergoing an allogeneic HSCT.^9^ Cutaneous 4 stage GvHD was treated with soft silicone dressings in addition to polyurethane foam dressings, leading to healing after 10 days of treatment. The dressing allowed pain control, wound healing, and the prevention of infections and skin maceration.^9^ However, our case was complex due to the development of the TMA that could have influenced the course of acute GvHD. When the immunosuppressive therapy was initially reduced with the suspension of cyclosporine and later of tacrolimus, the cutaneous GvHD reappeared, revealing the difficulty in treating these 2 complications simultaneously. However, the overall outcome was positive.

## CONCLUSIONS

Our case study contributes to the knowledge available regarding the management of acute cutaneous GvHD. In addition to systemic therapy as the gold standard, the use of an advanced dressing based on a soft silicone layer for wound care improved Mr T’s outcome. Primary well-designed studies are required to assess the effectiveness of soft silicone layers among the allogeneic HSCT recipients with cutaneous GvHD on a large scale to provide further recommendations for use.

## References

[1] Akpinar S Kayikci O Tekgunduz E. Defibrotide combined with triple therapy including posttransplant cyclophosphamide, low dose rabbit anti-t-lymphocyte globulin and cyclosporine is effective in prevention of graft versus host disease after allogeneic peripheral blood stem cell transplantation for hematologic malignancies. Transfus Apher Sci. 2022;61(1):103367. doi:10.1016/j.transci.2022.103367. 35120825

[2] Penack O Abouqateb M Peczynski C ATG or post-transplant cyclophosphamide to prevent GVHD in matched unrelated stem cell transplantation? Leukemia. 2024;38(5):1156-1163. doi:10.1038/s41375-024-02225-7. 38538862 PMC11073959

[3] Pawełczak-Szastok M Ilska M Swoboda R Giebel S. Trajectories of quality of life during hematopoietic stem cell transplantation: longitudinal cohort study. Sci Rep. 2025;15(1):5142. doi:10.1038/s41598-025-88748-0. 39934268 PMC11814371

[4] Zhang MY Zhao P Zhang Y Wang JS. Efficacy and safety of ruxolitinib for steroid-refractory graft-versus-host disease: systematic review and meta-analysis of randomised and non-randomised studies. PLoS One. 2022;17(7):e0271979. doi:10.1371/journal.pone.0271979. 35905125 PMC9337651

[5] Harris AC Young R Devine S International, multicenter standardization of acute graft-versus-host disease clinical data collection: a report from the Mount Sinai Acute GVHD International Consortium. Biol Blood Marrow Transplant. 2016;22(1):4-10.doi:10.1016/j.bbmt.2015.09.001. 26386318 PMC4706482

[6] Zeiser R Blazar BR Longo DL. Acute graft-versus-host disease - biologic process, prevention, and therapy. N Engl J Med. 2017;377(22):2167-2179. doi:10.1056/NEJMra1609337. 29171820 PMC6034180

[7] Mushtaq MU Shahzad M Tariq E Outcomes with mismatched unrelated donor allogeneic hematopoietic stem cell transplantation in adults: a systematic review and meta-analysis. Front Oncol. 2022;12:1005042. doi:10.3389/fonc.2022.1005042. 36276084 PMC9583270

[8] Gargiulo G Errico A De Cecco V Botti S Orlando L. La Gestione Infermieristica Della GVHD Acuta Cutanea. In: GITMO, ed. Handbook Vol. I, Sezione Infermieri. Italia: GIIMA; 2015:39-44.

[9] Vendlinski S. Relieving the pain of stage 4 graft-versus-host disease of the skin: the role of soft silicone dressing as an adjunct to medical management. Ostomy Wound Manage. 2004;50(12):14-18. 15632451

[10] Kim YJ Lee GH Kwong BY Martires KJ. Evidence-based, skin-directed treatments for cutaneous chronic graft-versus-host disease. Cureus. 2019;11(12):e6462. doi:10.7759/cureus.6462. 32025391 PMC6977575

[11] Chaganti P Gordon I Chao JH Zehtabchi S. A systematic review of foam dressings for partial thickness burns. Am J Emerg Med. 2019;37(6):1184-1190. doi:10.1016/j.ajem.2019.04.014. 31000315

[12] Penack O Marchetti M Aljurf M Prophylaxis and management of graft-versus-host disease after stem-cell transplantation for haematological malignancies: updated consensus recommendations of the European Society for Blood and Marrow Transplantation. Lancet Haematol. 2024;11(2):e147–e159.doi:10.1016/S2352-3026(23)00342-3. 38184001

[13] Rostagno E Campanati A Mordini N Phototherapy and topical treatments for cutaneous graft vs. host disease in haematopoietic stem cell transplantation patients: a scoping review. J Eur Acad Dermatol Venereol. 2022;36(7):1003-1015.doi:10.1111/jdv.18074. 35279894

[14] Campbell J Gavin N Button E Roberts N. Skin and wound care for individuals with graft versus host disease: a scoping review protocol. BMJ Open. 2020;10(10):e038567. doi:10.1136/bmjopen-2020-038567. PMC7545636 33033094

[15] Neumann J. Nursing challenges caring for bone marrow transplantation patients with graft versus host disease. Hematol Oncol Stem Cell Ther. 2017;10(4):192-194. doi:10.1016/j.hemonc.2017.06.001. 28683255

[16] Heyneman A Hoeksema H Vandekerckhove D Pirayesh A Monstrey S. The role of silver sulphadiazine in the conservative treatment of partial thickness burn wounds: a systematic review. Burns. 2016;42(7):1377-1386. doi:10.1016/j.burns.2016.03.029. 27126813

[17] Meaume S Senet P Dumas R Carsin H Pannier M Bohbot S. Urgotul: a novel non-adherent lipidocolloid dressing. Br J Nurs. 2002;11(16 Suppl):S42–S50. doi:10.12968/bjon.2002.11.Sup3.10556. 12362152

[18] David F Wurtz JL Breton N A randomised, controlled, non-inferiority trial comparing the performance of a soft silicone-coated wound contact layer (Mepitel One) with a lipidocolloid wound contact layer (UrgoTul) in the treatment of acute wounds. Int Wound J. 2018;15(1):159-169.doi:10.1111/iwj.12853. 29205809 PMC7949658

[19] Chen Y Zhao X Wang X Li LJ Wu L. The management of chronic graft-versus-host disease skin ulcers after hematopoietic stem cell transplantation: a case report. Adv Skin Wound Care. 2024;37(4):1-6. doi:10.1097/ASW.0000000000000122. 38506587

[20] Miura Y Nakagomi S. Management of cutaneous manifestations of genetic epidermolysis bullosa: a multiple case series. J Wound, Ostomy Continen Nurs. 2021;48(5):453-459. doi:10.1097/WON.0000000000000784. 34495939

[21] Williams G Withey S Walker CC. Longstanding pigmentary changes in paediatric scalds with a non-adherent siliconised dressing. Burns. 2001;27(2):200-202. doi:10.1016/s0305-4179(00)00082-6. 11226664

[22] Meuleneire F. Using a soft silicone-coated net dressing to manage skin tears. J Wound Care. 2022;11(10):365-369. doi:10.12968/jowc.2002.11.10.26440. 12494827

[23] Holm D Schommer K Kottner J. Review of medical adhesive technology in the context of medical adhesive-related skin injury. J Wound, Ostomy Continen Nurs. 2024;51(5S Suppl 5):S9–S17. doi:10.1097/WON.0000000000001115. 39313962

[24] White R Morris C. Mepitel: a non-adherent wound dressing with Safetac technology. Br J Nurs. 2009;18(1):58-64. doi:10.12968/bjon.2009.18.1.93582. 19127235

[25] Constable S Spitzer M. The role of value analysis in pressure injury prevention: a quality improvement project. J Wound, Ostomy Continen Nurs. 2025;52(1):29-35. doi:10.1097/WON.0000000000001140. 39835998

